# Omitting Sentinel Lymph Node Biopsy in Elderly Patients: A Lost Opportunity?

**DOI:** 10.1245/s10434-021-09727-z

**Published:** 2021-03-03

**Authors:** Todd M. Tuttle, Jane Yuet Ching Hui, Jianling Yuan

**Affiliations:** 1 Division of Surgical Oncology, Department of Surgery, University of Minnesota, Minneapolis, MN USA; 2 Department of Radiation Oncology, University of Minnesota, Minneapolis, MN USA

Sun and colleagues recently published an important study in *Annals of Surgical Oncology* that evaluated the impact of avoiding sentinel lymph node (SLN) biopsy for elderly patients with estrogen receptor (ER)-positive, clinically node-negative breast cancer.^1^ The authors concluded that SLN biopsy can be safely omitted in elderly patients with T1, ER-positive breast cancer.

The stated purpose of their study was to evaluate the recommendations from the Choosing Wisely^©^ Foundation, which conclude that patients > 70 years of age with early-stage ER-positive breast cancer and clinically negative nodes can be safely treated without axillary staging (https://www.choosingwisely.org/wp-content/uploads/2016/07/SSO-Choosing-Wisely-List.pdf). The results of the Cancer and Leukemia Group B (CALGB) 9343 trial are frequently cited to support this recommendation.^2^ In CALGB 9343, patients >70 years of age with clinical T1N0, ER-positive breast cancer underwent lumpectomy and were randomized to tamoxifen versus tamoxifen plus radiotherapy. Radiotherapy was significantly associated with an 8% absolute reduction in 10-year locoregional recurrence (LRR), but not with survival.

Sun and colleagues identified 267 elderly patients with T1, ER-positive, clinically node-negative breast cancer; 23% of these patients had SLN metastases.^1^ Although SLN status did not affect the LRR rates in the study by Sun et al., the Early Breast Cancer Trialists’ Collaborative Group reported a substantially higher risk of LRR for node-positive breast cancer after lumpectomy.^3^ Additionally, the absolute reduction in LRR after radiotherapy was much higher for node-positive patients. Thus, the absolute benefit of radiotherapy for elderly patients with SLN metastases is likely higher than the observed 8% benefit reported in CALGB 9343. Likewise, the benefit from radiotherapy for patients without SLN metastases is likely lower than 8%. Therefore, SLN biopsy should be considered for elderly patients to determine the magnitude of benefit from radiotherapy. In fact, surgical axillary staging and confirmed pathological N0 status were required for inclusion in the PRIME II trial, which randomized elderly patients to endocrine therapy alone versus radiotherapy plus endocrine therapy.^4^ In 2015, more than a decade after the initial publication of CALGB 9343, almost two-thirds of elderly patients with clinical stage I, ER-positive breast cancer still received radiotherapy after lumpectomy in the US.^5^ Perhaps the performance of SLN biopsy can be used to stratify radiotherapy among healthy, elderly patients.

Another argument against performing SLN biopsy in elderly patients is increased morbidity. However, the rates of lymphedema and other complications have been reported among patients receiving lumpectomy and radiotherapy, *along with* SLN biopsy. The added risk attributable solely to the SLN biopsy is likely very low.

We contend SLN biopsy is a minimally invasive procedure, with few long-term adverse effects, that can be performed safely at the time of lumpectomy. The status of the SLN provides important prognostic information and may be used to de-escalate the use of radiotherapy among elderly patients with SLN-negative, ER-positive breast cancer. Thus, SLN biopsy affords an important opportunity for patients and physicians to ‘choose wisely’ regarding the role of radiotherapy after lumpectomy. The use of SLN should be discouraged among elderly women with substantial comorbidities and limited life expectancy.
